# Comprehensive analysis of fatty acid metabolism-related gene signatures for predicting prognosis in patients with prostate cancer

**DOI:** 10.7717/peerj.14646

**Published:** 2023-01-10

**Authors:** Hongbo Wang, Zhendong Liu, Yubo Wang, Dali Han, Yuelin Du, Bin Zhang, Yang He, Junyao Liu, Wei Xiong, Xingxing Zhang, Yanzheng Gao, Panfeng Shang

**Affiliations:** 1 Lanzhou University Second Hospital, Lanzhou, Gansu, China; 2 Department of Urology, Key Laboratory of Urological Diseases in Gansu Province, Lanzhou University Second Hospital, lanzhou, Gansu, China; 3 Department of Orthopaedic People’s Hospital of Zhengzhou University, Henan Provincial People’s Hospital, Zhengzhou, Henan, China; 4 School of Basic Medicine and Forensic Medicine, Henan University of Science & Technology, Luoyang, Henan, China

**Keywords:** Prostate cancer, Fatty acid metabolism, Prognostic model, Recurrence free survival, Immune infiltration

## Abstract

Fatty acid metabolism (FAM) is an important factor in tumorigenesis and development. However, whether fatty acid metabolism (FAM)-related genes are associated with prostate cancer (PCa) prognosis is not known. Therefore, we established a novel prognostic model based on FAM-related genes to predict biochemical recurrence in PCa patients. First, PCa sequencing data were acquired from TCGA as the training cohort and GSE21032 as the validation cohort. Second, a prostate cancer prognostic model containing 10 FAM-related genes was constructed using univariate Cox and LASSO. Principal component analysis and t-distributed stochastic neighbour embedding analysis showed that the model was highly effective. Third, PCa patients were divided into high- and low-risk groups according to the model risk score. Survival analysis, ROC curve analysis, and independent prognostic analysis showed that the high-risk group had short recurrence-free survival (RFS), and the risk score was an independent diagnostic factor with diagnostic value in PCa patients. External validation using GSE21032 also showed that the prognostic model had high reliability. A nomogram based on a prognostic model was constructed for clinical use. Fourth, tumor immune correlation analyses, such as the ESTIMATE, CIBERSORT algorithm, and ssGSEA, showed that the high-risk group had higher immune cell infiltration, lower tumour purity, and worse RFS. Various immune checkpoints were expressed at higher levels in high-risk patients. In summary, this prognostic model is a promising prognostic biomarker for PCa that should improve the prognosis of PCa patients. These data provide new ideas for antitumour immunotherapy and have good potential value for the development of targeted drugs.

## Introduction

Prostate cancer (PCa) is the second most frequent malignancy in men and the fifth leading cause of cancer-related death (Ge et al., 2020). With recent improvements in surgical techniques and the application of a comprehensive treatment, the prognosis of prostate cancer has significantly improved. However, approximately 15% of patients have a biochemical recurrence within 5 years after surgery, and this recurrence rate reaches 40% within 10 years (Li et al., 2017b). Biochemical recurrence often leads to tumour development and metastasis, which results in poor prognosis. Therefore, it is important to clarify the risk of biochemical recurrence in patients at an early stage.

Current indicators, such as PSA, Gleason score, and lymph node invasion, are widely used in the clinic to monitor and assess prostate cancer recurrence (Stephenson et al., 2005). What’s more, many scholars are devoted to the discovery of novel biomolecular markers. For example, PCA3 is abnormally highly expressed in prostate cancer cells, and it is related to the Gleason pathological grading of prostate cancer, which is a promising prognostic marker (Kretschmer & Tilki, 2017). PTEN deletion activates the PI3K-AKT signaling pathway in prostate cancer cells to promote disease progression (Wise, Hermida & Leslie, 2017). Although there are some molecular biomarkers used for the diagnosis and treatment of prostate cancer, one or several biomarkers are not sufficient to meet the requirements of diagnosis, treatment, and prognosis evaluations of prostate cancer due to its complex pathological mechanism. The latest NCCN guidelines for prostate cancer include multivariate prognostic models based on gene expression classification and digital pathology biomarkers in the assessment system for prognostic evaluation and treatment decisions (Schaeffer & Srinivas, 2022). Therefore, it is highly necessary to develop new prognostic models to accurately and conveniently predict the prognosis of patients with prostate cancer.

Lipid metabolism, especially the synthesis of fatty acids (FAs), is an important cellular process that converts nutrients into metabolic intermediates for cell membrane synthesis, energy storage, and the production of signaling molecules. Fatty acid metabolism (FAM) is an important metabolic phenotype of tumour cells, and it is involved in a variety of pathological processes in tumour formation. For example, most tumours have an abnormally activated lipid metabolism that allows them to synthesize, prolong and desaturate fatty acids to support proliferation (Vriens et al., 2019). FAM also plays an important role in the malignant progression of tumours, such as invasion and metastasis. For example, activation of the TGFβ signaling pathway in lung cancer cells leads to reduced fatty acid synthesis *via* the inhibition of ChREBP, and knockdown of FASN leads to reduced expression of E-cadherin, which enhanced invasion and metastasis of lung cancer cells (Jiang et al., 2015).

Extensive studies confirm that lipid signaling plays a regulatory role in vascular growth and remodeling of the immune microenvironment (especially macrophages), and these alterations promote tumour angiogenesis (Kazlauskas, 2015; Mendelson, Evans & Hla, 2014). The lipid-derived factor PGE2 also leads to the formation of a suppressive immune response locally in tumours by inducing increased IL-10 release. PGE2 also regulates tumour-associated macrophage polarization towards the M2 type to participate in the pathological process of tumours (Luan et al., 2015). Numerous studies investigated the role of FAM in malignant tumorigenesis and progression from the perspective of pathogenesis, but some researchers attempted to construct prognostic prediction models using genes related to FAM. For example, the prognostic model constructed by Peng et al. (2021) using FAM-related lncRNAs has good diagnostic value for the prognosis of colorectal cancer patients. Qi et al. (2019) constructed a clinical prognostic model of glioma patients using FAM-related genes and found that the model-constructed genes correlated with immune cells and immune checkpoints in the tumour immune microenvironment. However, whether FAM-related genes are associated with prostate cancer prognosis and whether a relevant prognostic model may be established are not clear.

To fill this gap, the present study established a prostate cancer prognosis model using FAM-related genes to aid in the prediction of prognosis and immunotherapy for prostate cancer patients. First, RNA sequencing data of prostate cancer patients were obtained from the TCGA database, and 489 patients were genotyped according to FAM-related genes. Second, the prognostic model was established, and the prognostic risk score of each patient was calculated. The results of the prediction model were exhibited using nomograms. Finally, differences in the immune microenvironment, copy number variation, tumour mutational burden, and immune checkpoints were compared between the high- and low-risk groups. In conclusion, the present study established the first prognostic model of FAM in PCa, and the results provide new evidence and ideas for the prognostic assessment and treatment of prostate cancer and offer a new perspective for exploring its metabolic mechanisms.

## Materials and Methods

### Data collection

Tissue sample sequencing data and corresponding clinical follow-up data were obtained from the public database The Cancer Genome Atlas (TCGA, https://portal.gdc.cancer.gov/), including 499 cases of prostate cancer tissue and 52 cases of normal tissue. After excluding samples with incomplete clinical information, such as lack of survival time and some clinical characteristics, 489 tumour samples were included. Specific clinical information is shown in Table S1. Microarray data from the GSE21032 dataset from the Gene Expression Omnibus (GEO, https://www.ncbi.nlm.nih.gov/geo/) contained the sequencing data of 218 prostate cancer tissues and matching clinical information. After excluding samples with incomplete clinical information, 140 cases were included. Specific clinical information is shown in Table S2. The two datasets were normalized to remove batch effects.

The TIMER database (https://cistrome.shinyapps.io/timer/) is a public database for studying the abundance of immune cell infiltration in tumour tissues (Li et al., 2017a). It uses RNA-Seq expression profile data to detect the infiltration of immune cells in tumour tissues, including B cells, CD4+ T cells, CD8+ T cells, neutrophils, macrophages, and dendritic cells. This database was used to examine the effect of model-related genes on tumour immune infiltration.

Genomics of Drug Sensitivity in Cancer (GDSC, https://www.cancerrxgene.org/) provides free and publicly available genomic data on tumour therapy (Yang et al., 2013). It is dedicated to the discovery of potential tumour therapeutic targets to improve tumour treatment, and it is the largest public database of isotypes worldwide. Variations in the tumour genome will affect clinical treatment efficacy, and different targets have different responses to drugs. Therefore, the present study used this database to obtain the sensitivity of prostate cancer cells to anti-tumour drugs and their potential therapeutic targets.

The Human Protein Atlas database (HPA) provides tissue and cellular distribution information for all 24,000 human proteins. The expression of fatty acid metabolism-related proteins involved in the model building was investigated in normal and tumour tissues/organs using the HPA database (Colwill & Gräslund, 2011).

### Identification of differentially expressed fatty acid metabolism-related genes and cluster analyses

Fatty acid metabolism-related genes were obtained according to earlier research results and the Molecular Signatures Database, as shown in Table S3. Differentially expressed fatty acid metabolism-related genes in prostate cancer tissues and normal tissues were analysed using the “limma package” in R software. We used the STRING online tool to construct the PPI network of differentially expressed fatty acid metabolism genes (Szklarczyk et al., 2021). To study the role of fatty acid metabolism-related genes in the pathological process of prostate cancer, we used the “Consensus Cluster Plus package” to divide patients into different subtypes based on fatty acid metabolism-related gene expression and survival data, where reps were 50 and pItem was 0.8. The “cluster Profiler package” and the “org.Hs.eg.db package” were used to annotate the differentially expressed genes between different types. |Log2fold change| > 0.58 and a false discovery rate (FDR) <0.05 were the cut-off criteria for differential analysis.

### Prognostic model construction and verification

First, differentially expressed fatty acid metabolism-related genes associated with RFS in prostate cancer patients were determined using univariate analysis, and filtering criteria were *P* < 0.05. Second, the genes related to fatty acid metabolism associated with RFS were used to construct a prognostic model using LASSO Cox regression analysis (glmnet package) (Tibshirani et al., 2012). The following risk score calculation method was used: risk score = (coefficient mRNA 1 × expression of mRNA 1) + (coefficient mRNA 2 × expression of mRNA 2) +… (coefficient mRNA n × expression of mRNA n). The risk score of each sample in the TCGA-prostate cancer dataset was calculated. All samples were divided into high-risk and low-risk groups according to the median value of the risk score. The Kaplan-Meier method was used to analyse the prognostic differences between the high- and low-risk groups of patients. Receiver operating characteristic (ROC) curves were drawn to assess the accuracy and effectiveness of the risk model in predicting the prognosis of prostate cancer patients. Principal component analysis (PCA) and t-distributed stochastic neighbour embedding (t-SNE) methods were used to detect whether the prognostic model had reliable clustering ability.

### Independence of fatty acid metabolism-related gene models for the prognostic diagnosis of patients with prostate cancer

To clarify whether the fatty acid metabolism-related gene prognostic model had independent diagnostic significance for the RFS of prostate cancer patients, we performed univariate and multivariate analyses on the TCGA dataset and GEO dataset. Relevant clinical prognostic factors included age, TNM stage, and metastasis. *P* < 0.05 was considered statistically significant.

### Nomogram

A nomogram shows a predictive model intuitively and effectively, and it may be used for multi-index joint diagnosis or prediction of disease onset or progression. Nomograms were drawn to visualize the prognostic diagnostic model of this study. The nomograms quantified the risk score according to the stages of different clinical features and predicted the time of disease-free survival according to the total score. Nomogram plotting and calibration were performed using the R package “rms”.

### Gene set enrichment analysis

Gene set enrichment analysis (GSEA) is an efficient and widely used tool for the biological functional annotation of a set of genes (Subramanian et al., 2005). We divided 489 prostate cancer samples into high- and low-risk groups based on prognostic risk scores and performed GSEA. The gene set database was set to “KEGG cell signalling pathways”. *P* value < 0.05, q-value < 0.25, and |NES| > 1 were considered significantly enriched.

### Immune analysis

To examine differences in immune infiltration and the tumour immune microenvironment between the high- and low-risk groups, we performed a series of analyses. First, the stromal score, immune score, estimate score, and tumour purity were evaluated for each sample using the ESTIMATE algorithm, and the differences in relevant scores between the two groups were compared (Siemers et al., 2017). Second, the CIBERSORT algorithm was used to evaluate differences in immune cell infiltration for each sample in the two groups (Yoshihara et al., 2013). Single-sample gene enrichment analysis (ssGSEA) is an immune infiltration estimation method that uses the GSVA, GSEABase, and limma packages of the R language to estimate the degree of immune infiltration by calculating the expression levels of 29 immune gene sets in a single sample (Hänzelmann, Castelo & Guinney, 2013). In this study, ssGSEA was used to quantify the abundance of immune cells in the two groups of samples. Immune checkpoint correlation analysis was also performed to clarify the relationship between the sample risk score and immune checkpoints between the two groups. Immune checkpoints were accessed from previous literature (Tang et al., 2021).

### Somatic mutation analysis

Somatic mutation information of prostate cancer samples was obtained from TCGA. The mutation profiles of patients in the high- and low-risk groups of prostate cancer patients were analysed using the “maftool” package (Mayakonda et al., 2018). Tumour mutational burden (TMB) is the total number of somatic gene mutations per million bases. All samples were divided into a high TMB group and a low TMB group according to the median TMB values. The association of TMB with patient prognosis was evaluated using survival analysis and the relationship of TMB with the model risk score.

### Cell culture

Prostate cancer cells (22RV1 and DU145) were obtained from the China National Biomedical Experimental Cell Resource Bank. The following culture conditions were used: 22RV1 cells were cultured with RPMI 1640 medium containing 10% fetal bovine serum. DU145 cells were cultured with MEM containing 10% fetal bovine serum. All cells were cultured in a 37 °C incubator with 5% CO2.

### Quantitative reverse transcription PCR

Total RNA was extracted from cells using a Total RNA Kit I (R6834-02, Omega, USA). RNA quality and purity were checked using a NanoDrop (Thermo, USA). cDNA was obtained by reverse transcription using a NovoScript Plus All-in-one 1st Strand cDNA Synthesis SuperMix kit (E047-01B; Novoprotein, Shanghai, China). RT-qPCR was used to detect the expression level of ARPC1B using a Novostart SYBR qPCR SuperMix Plus Kit (E096-01A; Novoprotein, Shanghai, China). The primer sequences required for the experiments are shown in Table S4. GAPDH was used as an internal reference, and the 2^−ΔΔCT^ calculation method was used.

### Statistical analysis

Data analyses and processing were performed using R software (version 4.1.1). The Mann-Whitney U test was used to detect differential genes between normal and tumour tissues. RFS survival analysis was performed using the Kaplan–Meier method. To evaluate whether the predictive model could be used as an independent prognostic factor, univariate and multivariate Cox analyses were performed. The Wilcoxon test was used to analyse differences in immune cell types and immune checkpoints between the two groups. *P* < 0.05 was considered significantly different.

## Results

### Fatty acid metabolism related DEGs between normal and tumour tissues

**Figure 1:**
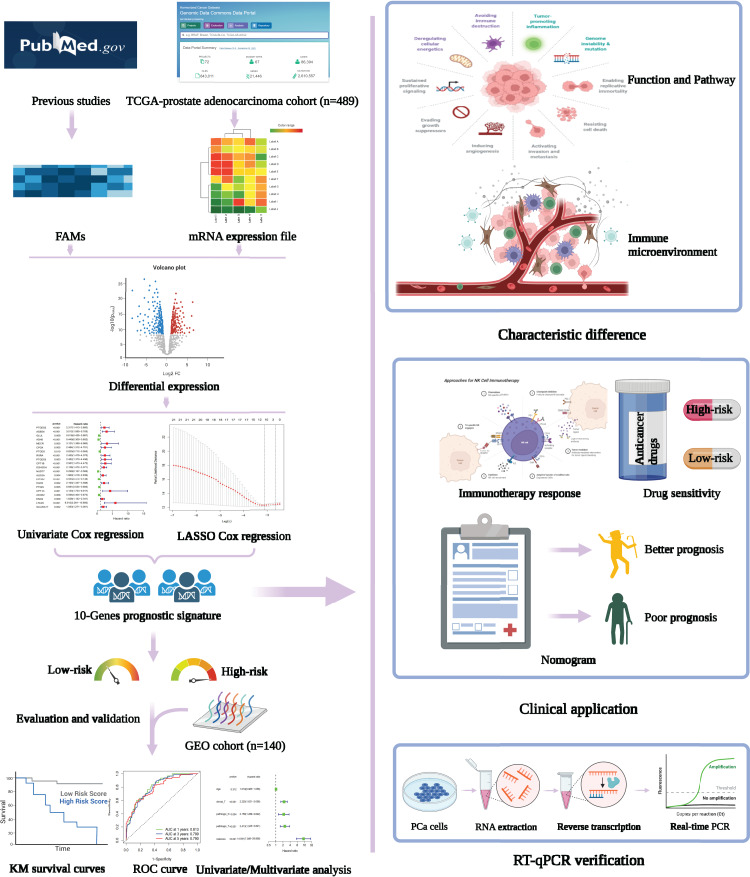
Flowchart presenting the process of establishing the gene signature and prognostic nomogram of PCa in this study. The flow chart was created with BioRender.com; All rights and ownership of BioRender content are reserved by BioRender.

**Figure 2:**
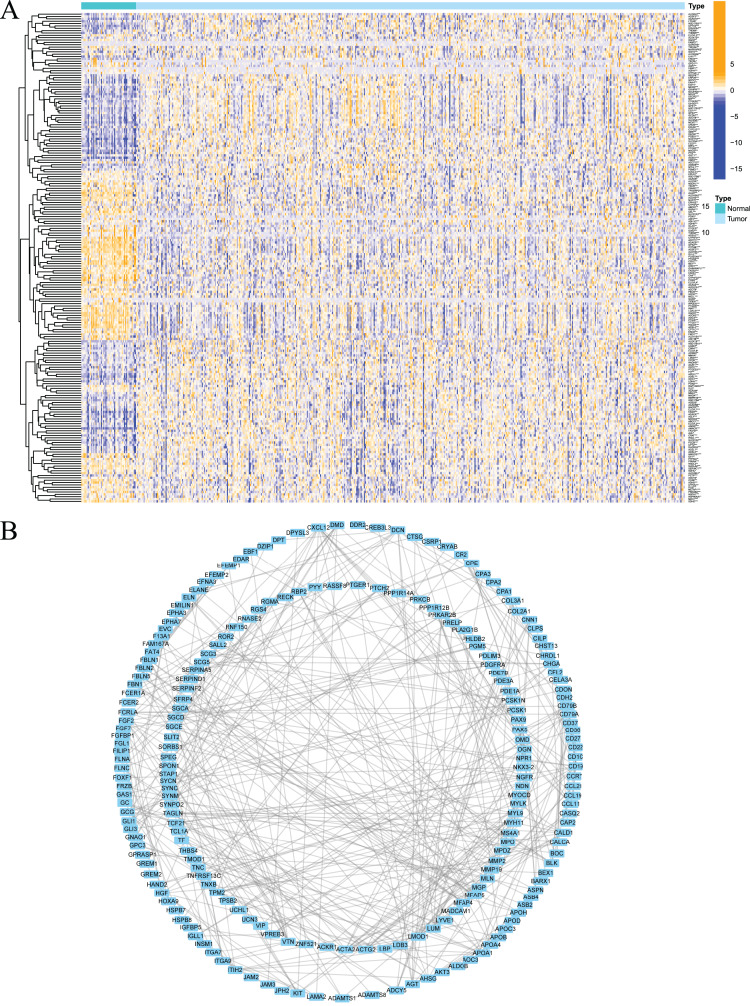
Expression of fatty acid metabolism-related genes in PCa tissues compared with normal kidney tissues and their interactions. (A) Heatmap of differentially expressed fatty acid metabolism related genes in prostate cancer and normal tissues from the TCGA dataset; (B) PPI network visualized using Cytoscape showed the interaction of fatty acid metabolism related genes. *P < 0.05, **P < 0.01, ***P < 0.001.

Firstly, the research flow chart of our study is shown in Fig. 1. The data for 489 prostate cancer samples were downloaded from the TCGA. To examine the biological function and role of fatty acid metabolism-related genes (FRGs) in the pathological process of prostate cancer, we performed gene differential expression analysis on tumour tissues and normal tissues obtained from the TCGA database. The differential expression results are shown in Fig. 2A. Blue shows low expression, and orange shows high expression. To examine the potential biological connection of these differentially expressed FRGs, we performed a PPI analysis. The results showed the interaction and relationship between the proteins encoded by these genes (Fig. 2B). These initial studies obtained the FAM-related DEGs and explored their connections.

### Clustering of tumours based on fatty acid metabolism-related genes

**Figure 3:**
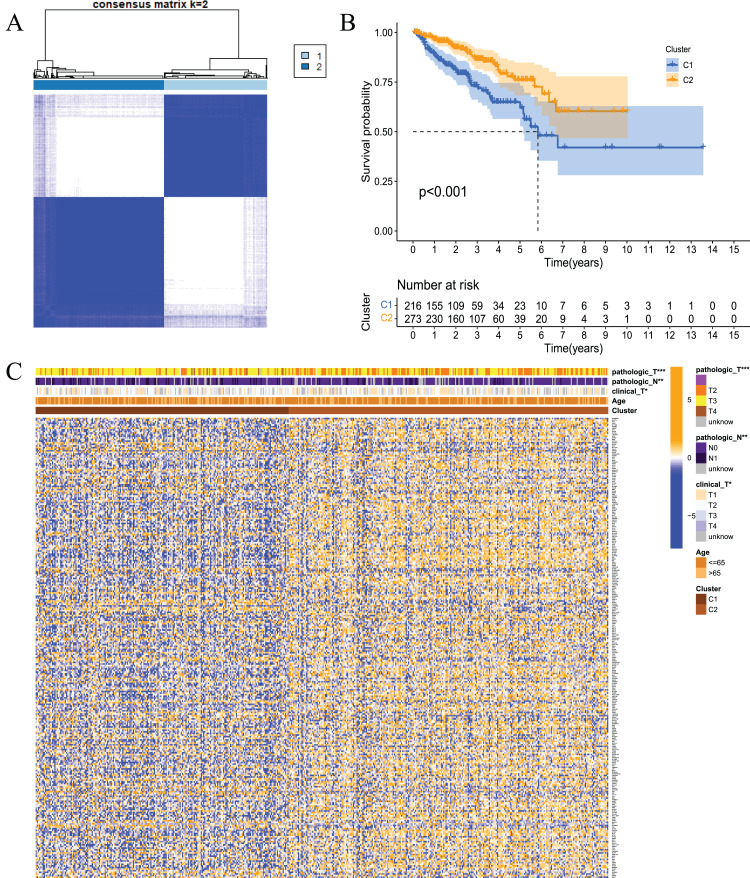
Tumour classification based on the fatty acid metabolism related DEGs. (A) Based on the results of the consensus clustering matrix (k = 2), 489 PCa patients were classified into two subtypes; (B) Kaplan–Meier survival analysis showed that there were differences in RFS between patients of the two subtypes; (C) Heatmap and the clinicopathologic characters of the two clusters classified by these DEGs. *P < 0.05, **P < 0.01, ***P < 0.001.

To examine whether prostate cancer may be divided into different subtypes based on FRGs, consensus clustering analysis was performed on 489 prostate cancer patients from TCGA. The results showed that the subgroup classification was stable and credible when the number of clusters was 2 (Fig. 3A). The 489 prostate cancer samples could be divided into two subtypes based on fatty acid metabolism-related genes. Survival analysis was performed to clarify the difference in the prognosis of the two subtypes of patients, and the results showed that C1 had a better prognosis than C2 (Fig. 3B). Cluster analysis was performed for the two subtypes. As shown in Fig. 3C, the gene expression patterns were different between the two subtypes, and the clinical features were significantly different, which indicated that FRG-based tumour classification was feasible.

### Construction of a prognostic model of prostate cancer based on fatty acid metabolism-related genes

**Figure 4:**
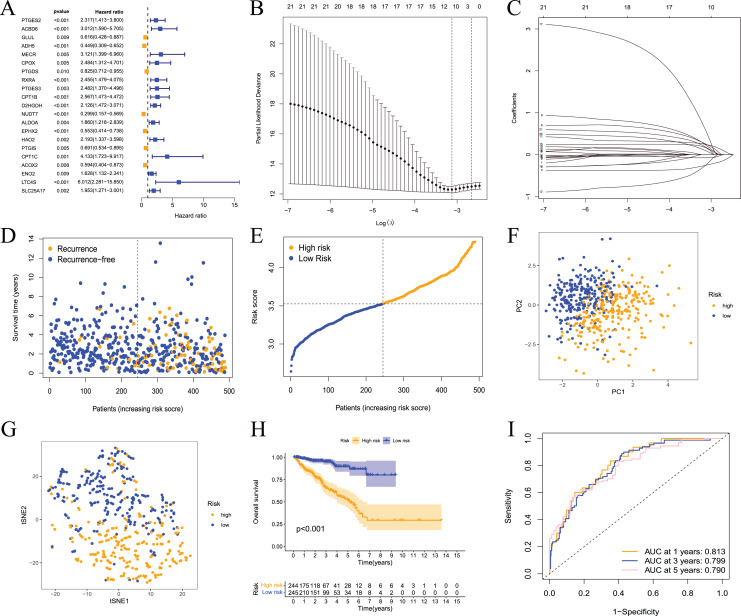
Prognostic risk model constructed based on TCGA training set. (A) Univariate Cox regression analysis identified fatty acid metabolism related DEGs associated with PCa prognosis; (B) cross-validation for tuning the parameter selection in the LASSO regression; (C) LASSO coefficient profiles of the 10 fatty acid metabolism related genes determined by the optimal lambda; (D) the distributions of recurrence free survival (RFS) status, RFS time, and risk score in the training set; (E) the distribution and median value of the risk scores in the training set; (F) PCA analysis showed that the two groups of patients had good discrimination; (G) tSNE analysis showed that the two groups of patients had a high degree of discrimination; (H) Kaplan–Meier survival curves showed a significant difference in RFS between the two groups of patients; (I) the ROC curve showed that the risk model had a high diagnostic value for prognosis.

These results showed that prostate cancer could be divided into two subtypes based on fatty acid metabolism-related genes. However, whether fatty acid metabolism-related genes were related to the RFS of prostate cancer patients and whether a prognostic model could be established were not known. Therefore, we performed univariate analysis for differentially expressed fatty acid metabolism-related genes, and 21 genes were related to the RFS of prostate cancer patients according to the shear criterion *P* < 0.01 (Fig. 4A). We performed least absolute shrinkage and selection operator (LASSO) regression analysis based on these genes. The results of the algorithm analysis (Figs. 4B and 4C) identified 10 candidate genes (ADH5, RXRA, PTGES3, D2HGDH, NUDT7, EPHX2, HAO2, CPT1C, LTC4S, and SLC25A17) that were selected for the construction of the prognostic model. The following risk score calculation formula was used: Risk score = (−0.25*ADH5 exp) + 0.25*RXRA exp + 0.22*PTGES3 exp + 0.24*D2HGDH exp + (−0.24*NUDT7 exp) + (−0.07*EPHX2 exp) + 0.16*HAO2 exp + 0.56*CPT1C exp + 0.09*LTC4S exp + (0.17*SLC25A17 exp). The risk score of 489 prostate cancer samples was calculated based on the formula. All samples were divided into high- and low-risk groups according to the median score (Figs. 4D and 4E). The PCA and tSNE analysis results showed that the distribution of patients in the two groups had mutual aggregation within the group, and there was good differentiation between the groups (Figs. 4F and 4G). This result indicated that the sample risk grouping had good reliability and discrimination. To clarify the difference in the RFS of the two groups of patients, we performed a survival analysis. The results showed that patients in the high-risk group had shorter recurrence-free survival and poor prognosis (Fig. 4H). The ROC curve showed that the area under the curve (AUC) values were 0.813, 0.799, and 0.790 at 1 year, 3 years, and 5 years, respectively (Fig. 3I). The clinical correlation analysis showed that the risk score increased with increasing clinical T, pathological T and N stages of prostate cancer, and the PSA and Gleason scores of patients in the high-risk group were higher than the low-risk group (Fig. S1). The expression level of the gene-encoded protein constructed by the model was verified using the HPA database (Fig. S2). These results showed that the prognostic risk model was dependable and had some diagnostic value for the prognosis of prostate cancer patients.

### Validation of the prognostic model

**Figure 5:**
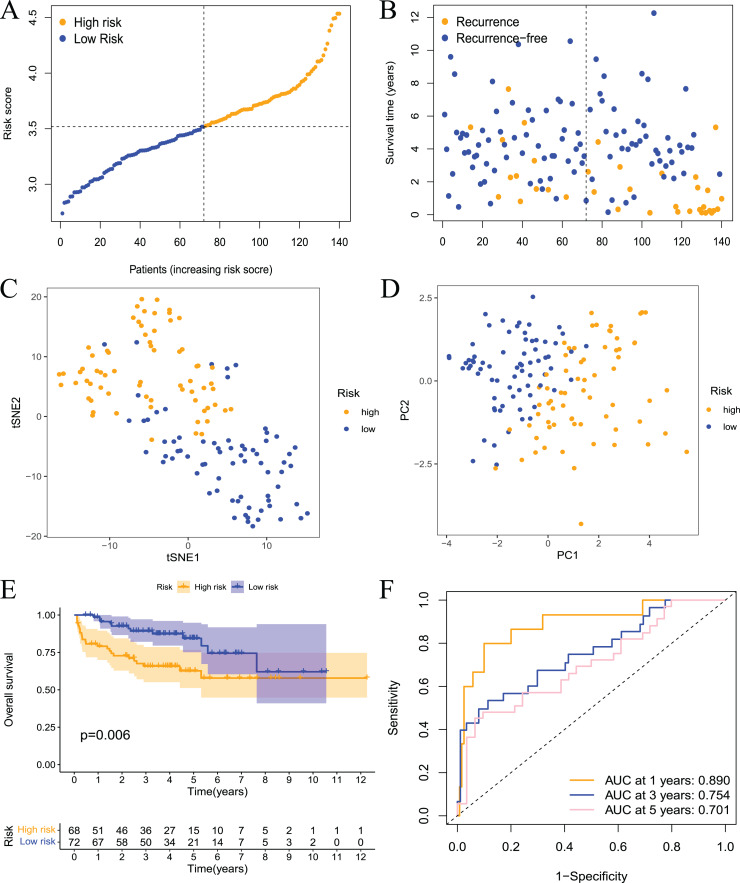
Validation of the risk model in the GEO dataset. (A) The distribution and median value of the risk scores in the validation set; (B) the distributions of RFS status, RFS time, and risk score in the validation set; (C) PCA analysis showed that the two groups of patients had good discrimination; (D) tSNE analysis showed that the two groups of patients had a high degree of discrimination; (E) Kaplan–Meier survival curves showed a significant difference in RFS between the two groups of patients; (F) the ROC curve showed that the risk model had a high diagnostic value for prognosis.

To further evaluate the validity and reliability of the prognostic model, the GSE21032 dataset containing 140 prostate cancer samples was used for external verification. First, the risk score of the sample in the GSE21032 dataset was calculated using the risk score calculation formula. The sample was divided into high- and low-risk groups based on the median risk score, and patients in the high-risk group had a larger recurrence ratio (Figs. 5A and 5B). Second, the PCA and tSNE analysis results showed good discrimination between the two groups of patients (Figs. 5C and 5D). This result indicated that the prognostic signature had high reliability. Survival analysis showed that the RFS of patients in the high-risk group was significantly worse (Fig. 5E). The ROC curve showed that the 1-, 3-, and 5-year AUC values were 0.890, 0.754, and 0.701, respectively (Fig. 5F.) This result indicated that the model had a certain diagnostic value for the RFS of prostate cancer patients in the GSE21032 dataset. These results showed that the prognostic risk model established in this study was effective for the external dataset GSE21032.

### The prognostic model is an independent prognostic factor in prostate cancer patients

**Figure 6:**
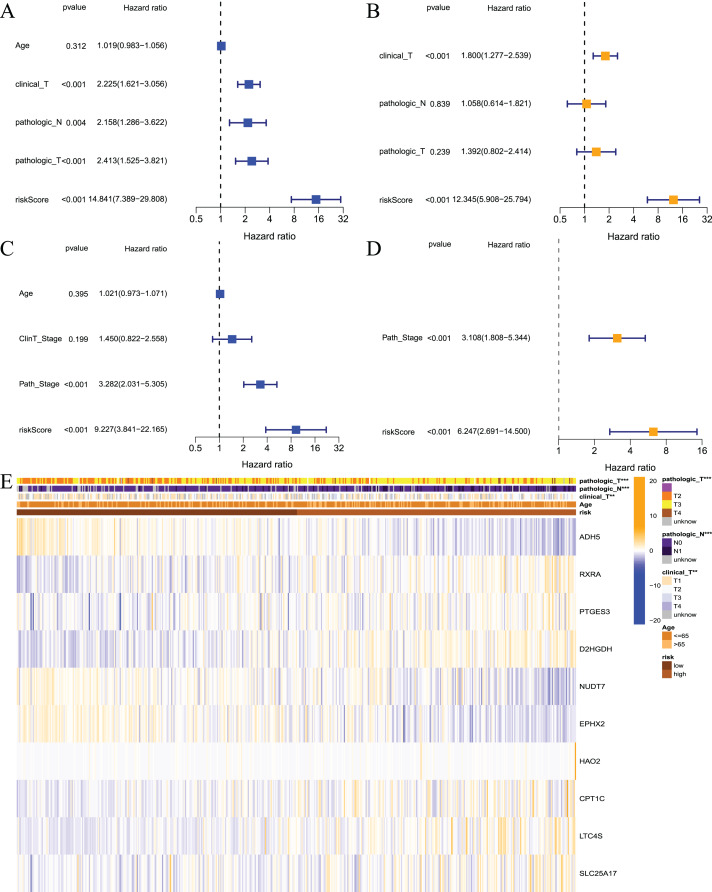
Independent prognostic analysis for risk model. (A) Univariate analysis based on TCGA dataset; (B) multivariate analysis based on TCGA database; (C) univariate analysis based on GEO dataset; (D) multivariate analysis based on GEO dataset; (E) heatmap for prognostic signature and clinicopathological manifestations. ***P < 0.001

The above results showed that the prognostic risk model established in this study was reliable and had a certain diagnostic value for the RFS of patients, but whether it could be used as an independent prognostic factor for prostate cancer patients was not known. Therefore, we performed univariate and multivariate regression analyses on patients from the TCGA and GEO datasets. The results of the univariate analysis showed that the risk score could be used as an independent prognostic factor in both datasets (Figs. 6A and 6C, TCGA: hazard ratio (HR) = 14.841, 95% confidence interval (CI) [7.389–29.808], *P* < 0.001; GEO: HR = 9.227, 95% CI [3.841–22.165], *P* < 0.001). Multivariate analysis also revealed that the risk score could be used as an independent prognostic factor in both datasets (Figs. 6B and 6D, TCGA: HR = 12.345, 95% CI [5.908–25.794], *P* < 0.001; GEO: HR = 6.247, 95% CI [2.691–14.5], *P* < 0.001). Cluster analysis was performed on model building genes and clinical features of the TCGA dataset. The results showed that the clinical characteristics of the two groups were significantly different (Fig. 6E). Briefly, these studies suggest that the model constructed in this study was an independent prognostic factor for prostate cancer patients.

### Construction of a predictive nomogram

**Figure 7:**
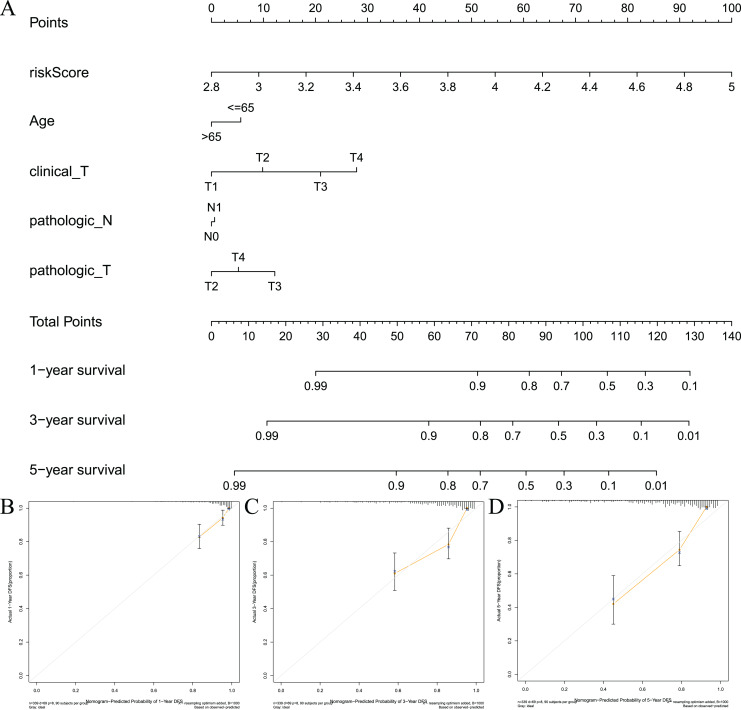
Constructing and validating nomograms. (A) Nomogram for predicting the 1-year, 3-year, and 5-year RFS of PCa patients; (B–D) calibration of the nomogram at 1-year, 3-year and 5-year survival.

To visualize the model, we constructed a model prediction nomogram (Fig. 7A). The nomogram included risk score, age, clinical T stage, pathological N stage, and pathological T stage. The nomogram was used to assess the prognosis of patients with prostate cancer. We performed graphic calibration to verify the validity of the nomogram. The x-axis in the calibration curve represents the nomogram-predicted recurrence rate of prostate cancer patients, and the y-axis represents the actual recurrence rate. The grey line represents the ideal predictive effect, and the orange line represents the actual predictive effect of the nomogram. As shown in Figs. 7B–7D, the coincidence of the two lines was higher, which indicated that the nomogram had a better prognostic prediction effect on patients’ 1-, 3-, and 5-year outcomes.

### Gene set enrichment analysis

**Figure 8:**
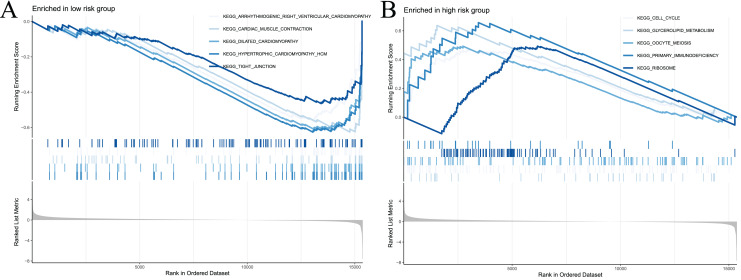
Significant enrichment results from GSEA analysis of two groups of patients. (A) The top five signaling pathways significantly enriched in the high-risk group; (B) the top five signaling pathways significantly enriched in the low-risk group.

To examine the potential differences in biological functions and cell signalling pathways between the high- and low-risk groups, we performed GSEA. According to the shear criteria (*P* value < 0.05 and q-value < 0.25), a total of 31 cell signalling pathways were enriched (Table S5). The top 5 pathways enriched in the high-risk group were “cell cycle”, “glycerolipid metabolism”, “oocyte meiosis”, “primary immunodeficiency” and “ribosome” (Fig. 8A). The top 5 pathways enriched in the low-risk group were “arrhythmogenic right ventricular cardiomyopathy”, “cardiac muscle contraction”, “dilated cardiomyopathy”, “hypertrophic cardiomyopathy” and “propanoate metabolism” (Fig. 8B). In addition to these partial immune-related pathways, multiple immune activity-related cell signalling pathways were enriched in the high-risk group, such as “ECM-receptor interaction”, “cytokine‒cytokine receptor interaction”, and “natural killer cell-mediated cytotoxicity”.

### Immune signature and immune checkpoint analysis

**Figure 9:**
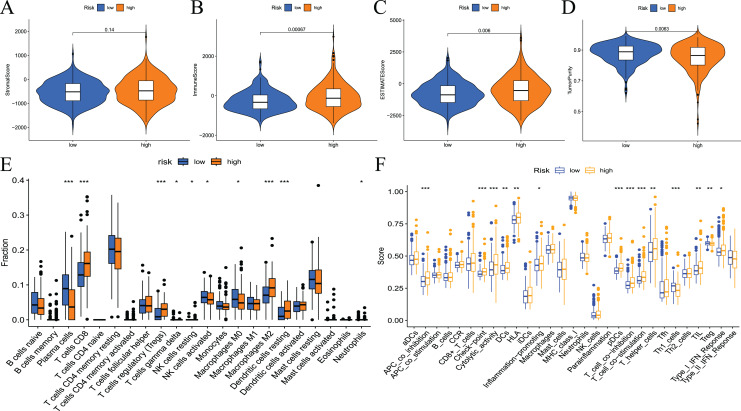
Differences in immune microenvironment and immune cell infiltration between high-and low-risk groups. (A–D) Comparisons between the two risk groups regarding stomal score, immune score, ESTIMATE score, and tumor purity; (E) the CIBERSORT algorithm quantified 22 immune cells between the high-risk and low-risk groups; (F) analysis of differences in immune cells and immune pathways between high- and low-risk groups of patients using ssGSEA.

To examine the correlation between prognostic models and immune characteristics, we performed a series of analyses. First, the ESTIMATE algorithm was used to clarify the differences in immune and stromal components in the immune microenvironment between the two groups of patients. The results showed that the ESTIMATE score, immune score, and stromal score of the high-risk group were higher than the low-risk group, and tumour purity exhibited the opposite results (Figs. 9A–9D). Survival analysis showed that patients with low tumour purity had a poor prognosis, and the remaining three immune scores negatively correlated with patient prognosis (Fig. S3). These results indicated that there were differences in the proportion of tumour and immune cells in the tumour microenvironment between high- and low-risk groups, which have implications for patient prognosis. The abundance of various immune cell infiltrates in the samples of the two groups is not known. Therefore, we used CIBERSORT for further analysis of the tumour immune microenvironment. The results are shown in Fig. 9E. The differences in plasma cells, CD8 T cells, regulatory T cells (Tregs), gamma delta T cells, resting NK cells, M0 macrophages, M2 macrophages, resting dendritic cells, and neutrophils were statistically significant. Notably, the immune cell score had a certain correlation with the model risk score. Survival analysis revealed that the expression levels of Tregs, CD8 T cells, plasma cells, activated NK cells, M2 macrophages, and resting dendritic cells negatively correlated with patient RFS (Fig. S4).

To assess the relationship between the risk score and immune status, the relevant immune cell gene set was quantified using ssGSEA. The results showed that the immune scores of APC co-stimulation, checkpoint, cytolytic activity, DCs, HLA, inflammation promotion, pDCs, T-cell co−inhibition, T-cell co−stimulation, T helper cells, Th1_cells, TILs, Tregs and type I IFN response were significantly different between the high- and low-risk groups (Fig. 9F). Survival analysis showed that most immune cells or function scores (Type I IFN response, TIL, T-cell co-inhibition, T helper cells, T-cell co-stimulation, pDCs, HLA, inflammation promotion, DCs, cytolytic activity, APC co-inhibition, and checkpoint) negatively correlated with the patient’s RFS. Treg and Th1 cells positively correlated with patient RFS (Fig. S5).

**Figure 10:**
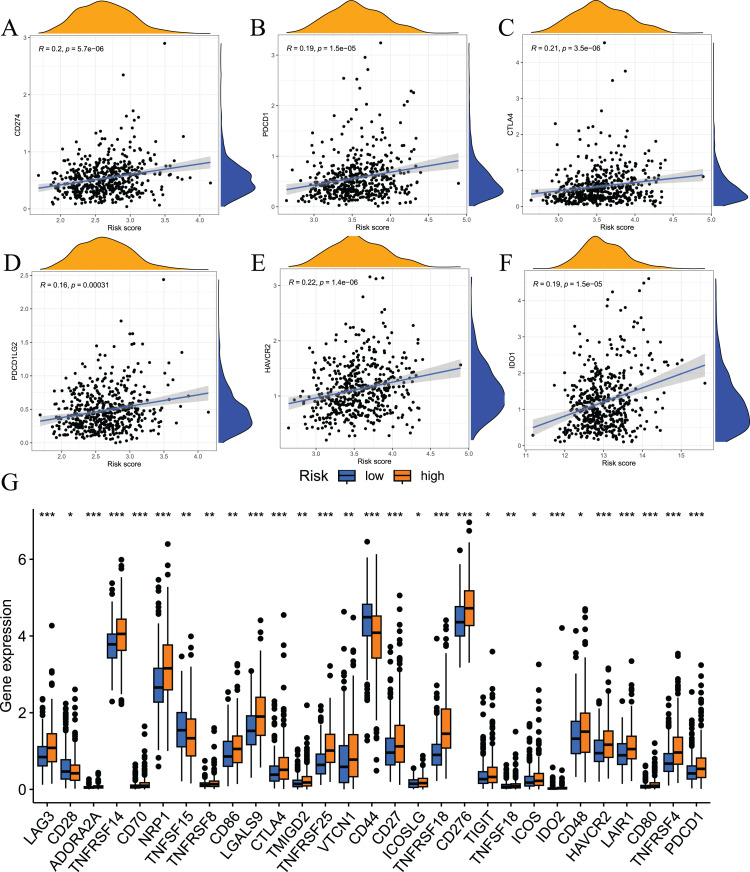
Results of immune checkpoint analysis. (A–F) correlation of immune checkpoints with risk score; (G) differences in expression levels of immune checkpoints between high and low risk groups.

Immune checkpoint inhibitors are an important breakthrough in the field of oncology. These inhibitors have been clinically used in a variety of solid tumours, and the effects are good. To examine the relationship between risk scores and immune checkpoints, we first performed a correlation analysis. The results showed that the expression levels of immune checkpoints, such as CD274, CTLA4, PDCD1, HAVCR2, PDCD1LG2, and IDO1, positively correlated with risk scores (Figs. 10A–10F). We also analysed the expression levels of other immune checkpoints in the two sets of samples. The results showed that the expression levels of the high-risk group were significantly higher than the low-risk group (Fig. 10G). Taken together, these findings suggest that the fatty acid metabolism risk model is a predictor of immunotherapy response.

### Tumour somatic mutation characteristics and the relationship between model-related gene copy number variation and immune infiltration

**Figure 11:**
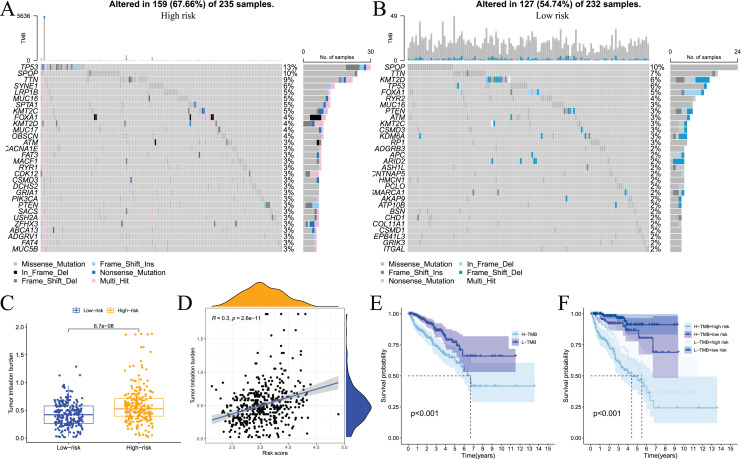
Analysis of tumor somatic cell mutations in two groups of patients with high and low risk. (A) The 30 genes most frequently mutated in the high-risk group; (B) the 30 genes most frequently mutated in the low-risk group; (C) differences in tumor mutation burden (TMB) between high - and low-risk groups; (D) correlation of risk score with TMB; (E) and (F) prognostic difference between high and low TMB and high and low risk in PCa patients.

Somatic mutations in the genome continue to accumulate during the pathological process of tumours to drive the occurrence and development of tumours. Tumour gene mutations are of great significance to tumour treatment, drug resistance monitoring, and prognostic evaluation. Therefore, we performed genetic mutation analysis on samples from the high- and low-risk groups, and the top 20 genes with the highest mutation frequency in the two groups are shown in Figs. 11A and 11B. We also performed an analysis of the relationship of TMB with the model risk score and prognosis. Differences in TMB between the high- and low-risk groups are shown in Fig. 11C. The high-risk group had significantly higher TMB than the low-risk group. The relationship between TMB and risk score was positively correlated (Fig. 11D). The survival analysis results showed that high TMB tended to have a poor prognosis (Fig. 11E and 11F). Taken together, these results indicate that there are differences in TMB between patients in the high- and low-risk groups and indirectly indicate that the two groups of patients respond differently to immunotherapy.

**Figure 12:**
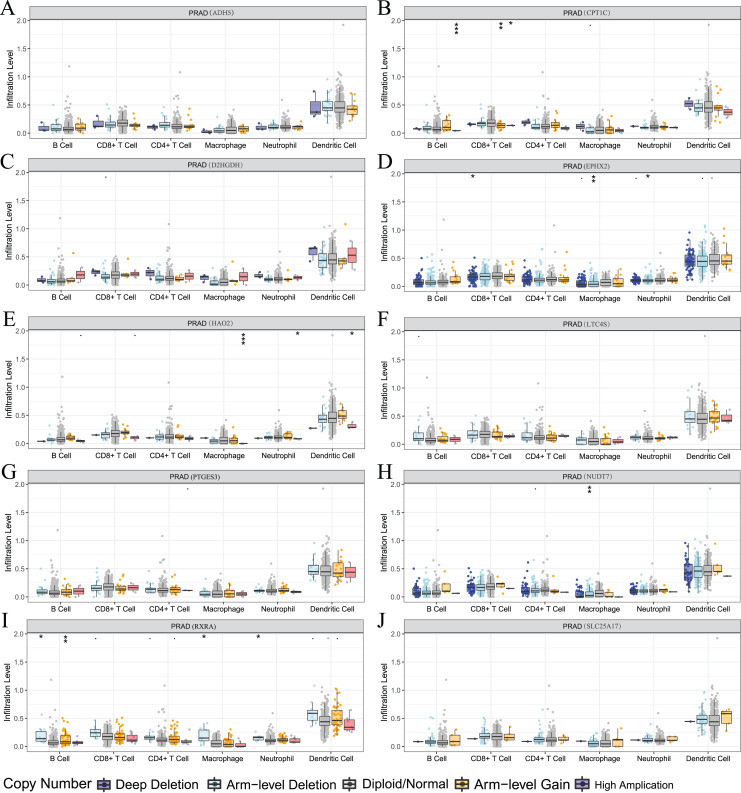
Relationship between signature genes and immune cell infiltration. (A) ADH5, (B) CPT1C, (C) D2HGDH, (D) EPHX2, (E) HAO2, (F) LTC4S, (G) NUDT7, (H) PTGES3, (I) RXRA and (J) SLC25A17. *P < 0.05, **P < 0.01, ***P < 0.001.

We also used the TIMER analysis tool to clarify the relationship between model-related gene copy number variation (CNV) and immune cell infiltration. The results showed that the CNVs of model-associated genes induced changes in the levels of partial immune cell infiltration. As shown in Fig. 12, deep deletion, arm-level deletion, and high amplification reduced the infiltration of some immune cells. We examined the relationship between the expression of model-related genes and the abundance of immune cell infiltration using TIMER, and the results showed a certain correlation. These results suggested that expression level is a potential factor affecting the immune microenvironment of prostate cancer (Fig. S6).

### Drug sensitivity analysis

**Figure 13:**
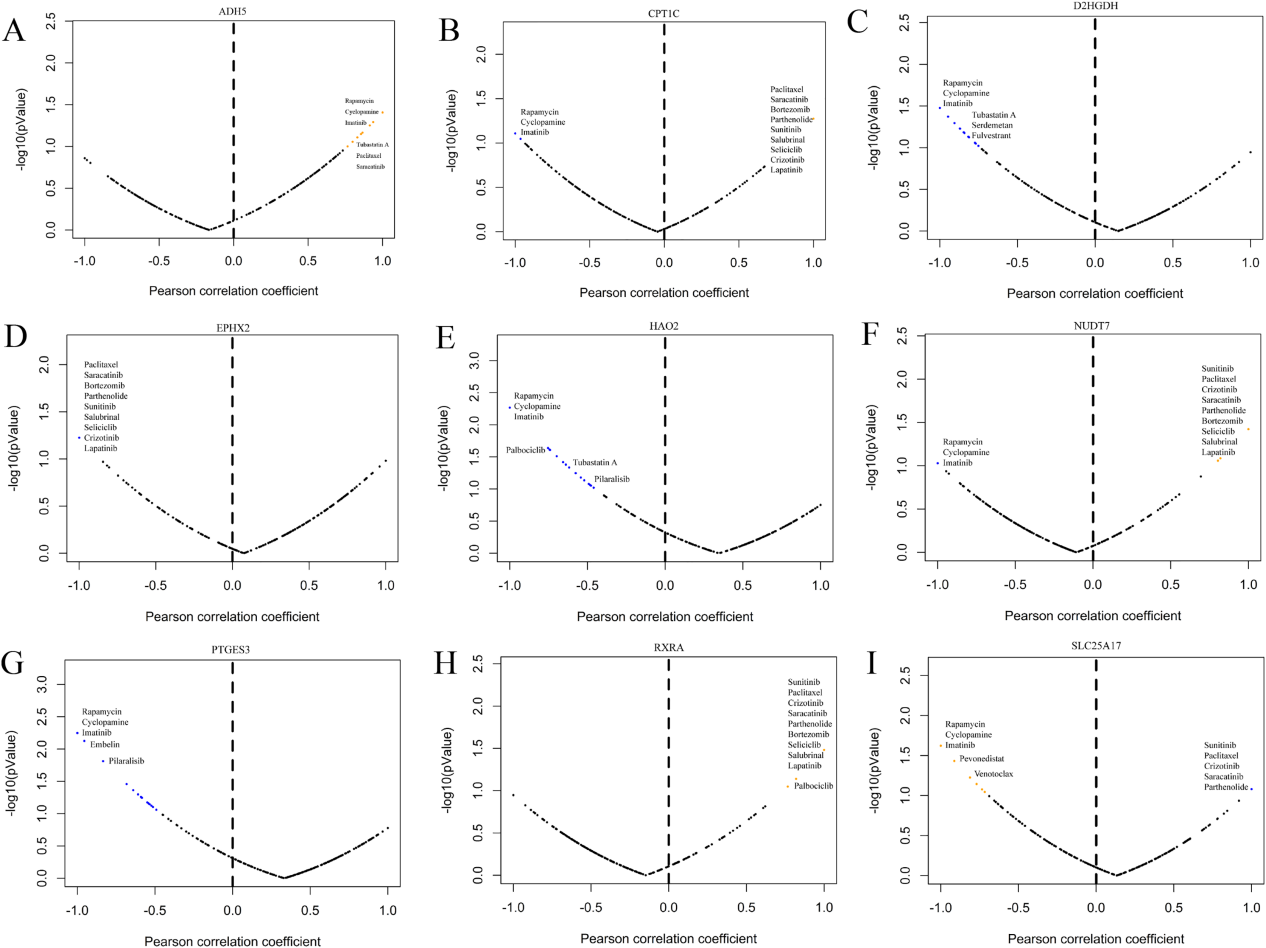
Correlation study between the expression of signature genes in PCa cell lines and partial antitumor drug sensitivity. (A) ADH5, (B) CPT1C, (C) D2HGDH, (D) EPHX2, (E) HAO2, (F) NUDT7, (G) PTGES3, (H) RXRA and (I) SLC25A17.

GDSC is a practical database containing the sensitivity and response of various tumour cells to drugs. To clarify whether the model-related genes were potential drug treatment targets for prostate cancer and which drugs were more sensitive to this target, we performed correlation analysis. The results showed that model-related genes in prostate cancer cells were related to the drug resistance or sensitivity of some tumour-targeted drugs (Fig. 13). The expression of CPT1C, D2HGDH, HAO2, LTC4S NUDT7, PTGES3 and SLC25A17 negatively correlated with the sensitivity to rapamycin and imatinib. CPT1C, LTC4S, NUDT7 and RXRA positively correlated with sensitivity to lapatinib and salubrinal. The expression of CPT1C, LTC4S, NUDT7, RXRA and SLC25A17 positively correlated with sunitinib sensitivity. These results provide new potential ideas for targeted therapy of prostate cancer.

### Verification of the expression of the model construction gene in PCa cells

**Figure 14:**
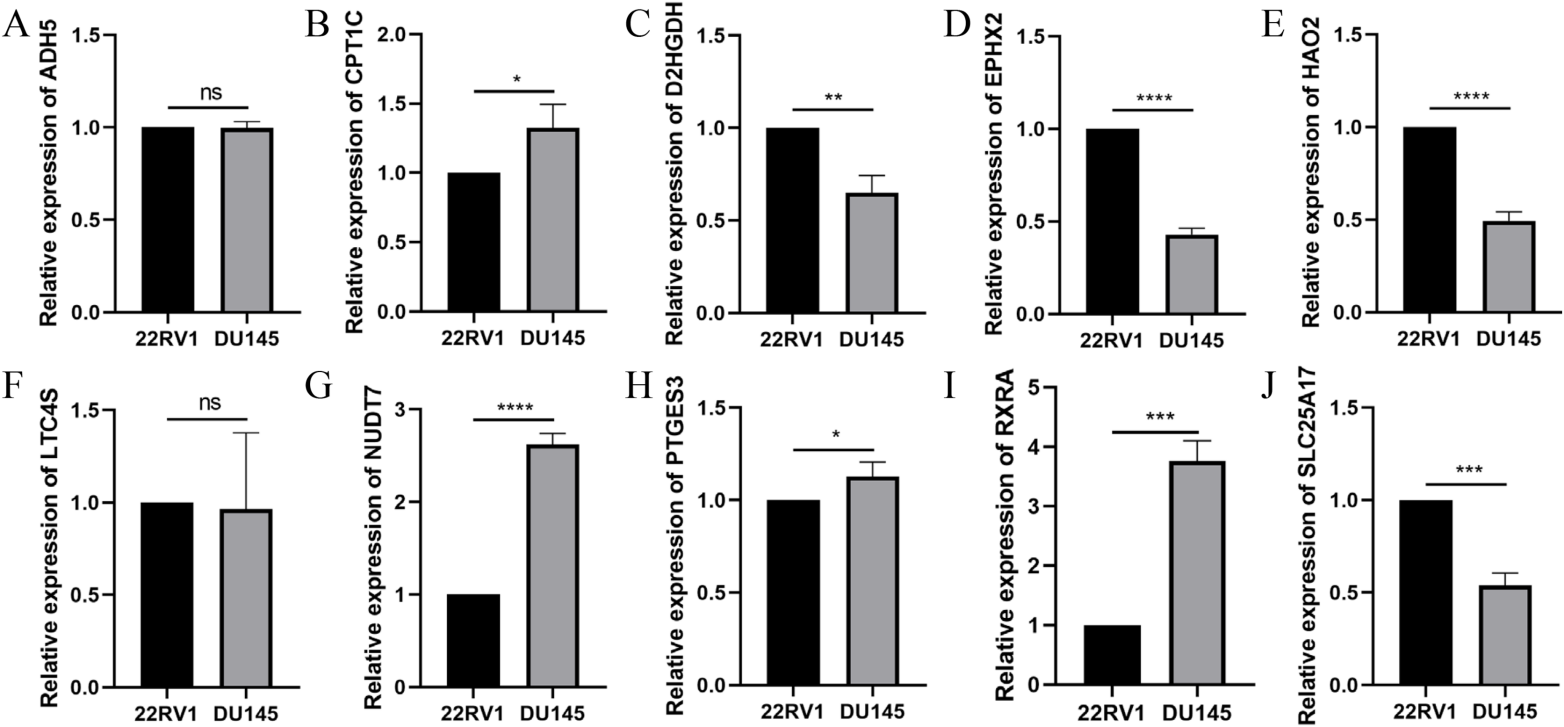
Validation of the expression of signature genes in PCa cell lines. (A) ADH5, (B) CPT1C, (C) D2HGDH, (D) EPHX2, (E) HAO2, (F) LTC4S, (G) NUDT7, (H) PTGES3, (I) RXRA and (J) SLC25A17. *P < 0.05, **P < 0.01, ***P < 0.001, ****P < 0.0001.

To further validate the effectiveness of the signature, we used the primary human prostate cancer cell line 22RV1 and the human prostate cancer metastasis-derived cell line DU145 as the study subjects and detected the expression levels of the signature in prostate cancer cells of different aggressiveness levels using RT-qPCR. As presented in Fig. 14, CPT1C, NUD7T, PTGES3 and RXRA were elevated in DU145 cells compared to 22RV1 cells. D2HGDH, EPHX2, HAO2 and SLC25A17 were expressed at low levels in DU145 cells. Taken together, these model genes showed significant differences in PCa cell lines, which indirectly confirmed the importance and validity of these model genes in PCa.

## Discussion

Metabolic disorders are well recognized as independent risk factors affecting the prognosis of prostate carcinogenesis and metastatic prostate cancer (Gacci et al., 2017). FAM is a key metabolic pathway in the regulation of immune responses. FAM provides energy for immune cells and substrates and precursors for the synthesis of various cellular components and signalling molecules (Li & Zhang, 2016). Enhanced *de novo* synthesis of fatty acids is a hallmark metabolic abnormality of tumour cells, and it is involved in tumour cell transformation, proliferation and migration (Jafari et al., 2019). Therefore, enzymes related to FAM have become reasonable targets for anti-tumour therapy and markers for prognosis evaluation (Fhu & Ali, 2020). However, the value of FAM-related genes for prostate cancer diagnosis, treatment, and prognosis evaluation has not been fully elucidated. Therefore, it is necessary to establish an integrated diagnostic model for prostate cancer prognosis based on FAM-related genes.

The present study first examined the expression levels of FAM-related genes in prostate cancer tissue, and the results showed that most of the FAM-related genes were differentially expressed in prostate cancer tissue and normal tissue. This result also showed that FAM-related genes had potential effects on the pathological process of prostate cancer. Prostate cancer was divided into two subtypes based on FAM-related gene expression. Further clustering analysis of survival analysis showed that patients in the two subtypes had obvious differences in prognosis and some differences in some clinical characteristics between patients in the two subtypes. To deeply evaluate the value of FAM-related genes for the prognosis of prostate cancer patients, we constructed a prognostic model by screening genes related to prognosis as candidates using univariate Cox analysis. We constructed a prognostic model that included 10 FAM-related genes using the LASSO algorithm. Prostate cancer patients were divided into high- and low-risk groups according to the model. Principal component analysis showed that the prognostic model had high reliability and discrimination between high- and low-risk patients. The results of survival, ROC, univariate, and multivariate analyses showed that the risk score model was an independent prognostic risk factor and had better prognostic diagnostic significance. Notably, the results of clinical correlation analysis showed that the risk score increased with higher T stage and N stage and positively correlated with Gleason score and PSA level. The risk score was closely related to these traditional prognostic indicators, which also indirectly supports the diagnostic value of the prognostic model for patient prognosis. To verify the validity of the model, the present study used an external dataset for validation, which gave better results. To verify the effectiveness of the model, the present study used an external dataset for verification and obtained good results. These results show that the model effectively predicted the prognosis of prostate cancer patients and provide help for the diagnosis and treatment of prostate cancer patients.

The prognostic model in the present study consisted of 10 genes, including ADH5, RXRA, PTGES3, D2HGDH, NUDT7, EPHX2, HAO2, CPT1C, LTC4S and SLC25A17. ADH5, NUDT7 and EPHX2 expression was decreased in the high-risk group, and RXRA, PTGES3 D2HGDH, HAO2, CPT1C, LTC4S and SLC25A17 were increased in the high-risk group. Previous studies confirmed that EPHX2, RXRA, LTC4S and SLC25A17 were abnormally expressed in prostate cancer tissues and affected the malignant biological behaviour of prostate cancer cells and prognosis of patients (Ali et al., 2018; Liu et al., 2021; Kushwaha et al., 2022; Xue et al., 2020). ADH5, NUDT7, PTGES3, D2HGDH, HAO2 and CPT1C also play regulatory roles in the pathological processes of various tumours (Adekeye et al., 2022; Han et al., 2018; Song et al., 2020; Li et al., 2021; Gaudet et al., 2011; Xiao et al., 2019). Taken together, these findings suggest that each gene involved in model building plays an important role in prostate cancer or other tumour pathological processes. These findings revealed that the prognostic model constructed in this study had a better effect. The tumour immune microenvironment is a complex and tightly integrated whole, and the components interact to regulate the invasive progression of tumours *via* various mechanisms, such as induction of immunosuppression and promotion of angiogenesis (Hinshaw & Shevde, 2019). However, whether the genes in the model construction play a role in the pathological process of prostate cancer *via* regulation of the tumour immune microenvironment is not known.

Many scholars have begun to focus on the role of the tumour microenvironment in tumour progression. The tumour immune microenvironment plays an important role in the development and malignant progression of prostate cancer by inducing cancer cell proliferation, enhancing cancer anti-apoptotic pathways, and stimulating angiogenesis to promote cancer spread (Shiao, Chu & Chung, 2016). The potential differences in the tumour microenvironment between the high- and low-risk groups were not known. Therefore, we performed a correlation analysis of tumour immunity. First, the results of ESTIMATE algorithms showed that the high-risk group had a higher immune score and lower tumour purity compared to the low-risk group. The results of survival analysis showed that patients with high immune scores had a poor prognosis, and patients with high tumour purity had a better prognosis. The results of this study are consistent with previous studies. For example, Sun et al. (2021) used the ESTIMATE algorithm to demonstrate that renal cancer patients with high immune scores tended to have a better prognosis than patients with low immune scores. Zhang et al. (2017) showed that low tumour purity was an important factor that promoted the malignant biological behaviour of glioma cells and led to poor patient prognosis. This study performed the CIBERSORT algorithm and ssGSEA immune correlation analysis based on a prognostic model and showed that high risk scores were associated with a poor prognosis and an increased infiltration of multiple immune cells. The results of studies on immune infiltration in gliomas showed that overall survival was poorer in patients with tumours with high immune infiltration status (Yin et al., 2020). These findings suggest that fatty acid metabolism-related genes involved in the construction of prognostic models regulate the tumour microenvironment of prostate cancer and play an important role in its pathological process.

Tumour immunotherapy is one of the most successful approaches to cancer treatment in recent years. Many recent scholars and clinicians have begun to focus on the effectiveness of immune checkpoint inhibitors in patients with prostate cancer. We investigated the relationship between model risk scores and immune checkpoint expression levels. The results showed a difference in immune checkpoint expression levels between the two groups of patients (29/29), with relatively higher expression levels in the high-risk group. This result indicates that patients in the high-risk group may be more sensitive to immune checkpoint inhibitors. The present study also analysed the effect of copy number variation in prostate cancer. The results showed that tumour mutational burden positively correlated with the model risk score, and patients with high mutational burden tended to have poor prognosis. These results provide a new perspective for the study of the prostate cancer tumour microenvironment. Overall, the findings of the present study indicate that the prognostic risk model is valuable for the prognosis of prostate cancer patients and the evaluation of tumour immune infiltration status.

Although the prognostic model constructed in this study using transcriptome data and patient follow-up information of large samples from two public databases had good validity, it failed to deeply validate the model for clinical use in a large sample, multicenter, prospective study. This factor is a limitation of this study and a direction for our future efforts.

## Conclusions

The present study constructed a FAM-related prognostic model for prostate cancer based on FAM-related genes that exhibited high predictive power. We examined differences in immune infiltration between low- and high-risk prostate cancer patients, and the results provide some evidence for targeted therapy and immunotherapy of prostate cancer. To the best of our knowledge, this study is the first study to construct a fatty acid metabolism-related prognostic model for prostate cancer. Therefore, the results provide new insights for examining the molecular mechanism and prognostic evaluation of prostate cancer.

## Supplemental Information

10.7717/peerj.14646/supp-1 Supplemental Information 1 Differences in risk scores for different clinical characteristics.

10.7717/peerj.14646/supp-2 Supplemental Information 2 Detection of protein expression levels encoded by model genes based on the Human Protein Atlas.

10.7717/peerj.14646/supp-3 Supplemental Information 3 Relationship between risk score and ESTIMATE score (A–D); Prognostic differences between high and low ESTIMATE scores (E–H).

10.7717/peerj.14646/supp-4 Supplemental Information 4 Relationship of different immune cells and risk scores (A–F); Prognostic impact of differences in immune cell infiltrate abundance (G–L).

10.7717/peerj.14646/supp-5 Supplemental Information 5 The effect of different immune activities on the prognosis of patients.

10.7717/peerj.14646/supp-6 Supplemental Information 6 Correlation of model gene expression levels with the abundance of immune cell infiltration. Correlation of model gene expression levels with the abundance of immune cell infiltration.

10.7717/peerj.14646/supp-7 Supplemental Information 7 Characteristics of patients with prostate cancer based on TCGA.

10.7717/peerj.14646/supp-8 Supplemental Information 8 Characteristics of patients with prostate cancer based on GEO.

10.7717/peerj.14646/supp-9 Supplemental Information 9 List of genes related to fatty acid metabolism.

10.7717/peerj.14646/supp-10 Supplemental Information 10 Primer sequence information of RT qPCR.

10.7717/peerj.14646/supp-11 Supplemental Information 11 Specific results of GSEA analysis.

10.7717/peerj.14646/supp-12 Supplemental Information 12 Results of RT-qPCR detection of expression levels of model building genes in PCa cell lines (LTC4S).

10.7717/peerj.14646/supp-13 Supplemental Information 13 Results of RT-qPCR detection of expression levels of model building genes in PCa cell lines (ADH5, CPT1C, D2HGDH, EPHX2, HAO2, NUDT7, PTGES3, RXRA, and SLC25A17).

## ABBREVIATIONS

FAM: Fatty acid metabolism
PCa: Prostate cancer
RFS: Recurrence-free survival
FAs: Fatty acids
TCGA: The Cancer Genome Atlas
GEO: Gene expression omnibus
GDSC: Genomics of Drug Sensitivity in Cancer
HPA: The Human Protein Atlas database
ROC: Receiver operating characteristic
PCA: Principal component analysis
t-SNE: t-distributed stochastic neighbor embedding
GSEA: Gene set enrichment analysis
TMB: Tumor mutational burden
LASSO: Least absolute shrinkage and selection operator
AUC: Area under the curve
HR: Hazard ratio
CI: Confidence interval
FRGs: Fatty acid metabolism related genes
Tregs: T cells regulatory
CNVs: copy number variation

## Additional Information and Declarations

### Data Availability

The following information was supplied regarding data availability: The data is available at NCBI GEO: GSE21032 and Zenodo: Hongbo Wang. (2022). Comprehensive analysis of fatty acid metabolism-related gene signatures for predicting prognosis in patients with prostate cancer. https://doi.org/10.5281/zenodo.7323463.

## References

[ref-1] Adekeye A, Agarwal D, Nayak A, Tchou J (2022). PTGES3 is a putative prognostic marker in breast cancer. Journal of Surgical Research.

[ref-2] Ali HEA, Lung PY, Sholl AB, Gad SA, Bustamante JJ, Ali HI, Rhim JS, Deep G, Zhang J, Elmageed ZYA (2018). Dysregulated gene expression predicts tumor aggressiveness in African-American. Scientific Reports.

[ref-3] Colwill K, Gräslund S (2011). A roadmap to generate renewable protein binders to the human proteome. Nature Methods.

[ref-4] Fhu CW, Ali A (2020). Fatty acid synthase: an emerging target in cancer. Molecules.

[ref-5] Gacci M, Russo GI, Nunzio CDe, Sebastianelli A, Salvi M, Vignozzi L, Tubaro A, Morgia G, Serni S (2017). Meta-analysis of metabolic syndrome and prostate cancer. Prostate Cancer and Prostatic Diseases.

[ref-6] Gaudet P, Livstone MS, Lewis SE, Thomas PD (2011). Phylogenetic-based propagation of functional annotations within the gene ontology. Brief Bioinform.

[ref-7] Ge R, Wang Z, Montironi R, Jiang Z, Cheng M, Santoni M, Huang K, Massari F, Lu X, Cimadamore A, Lopez-Beltran A, Cheng L (2020). Epigenetic modulations and lineage plasticity in advanced prostate cancer. Annals of Oncology.

[ref-8] Han J, Jackson D, Holm J, Turner K, Ashcraft P, Wang X, Cook B, Arning E, Genta RM, Venuprasad K, Souza RF, Sweetman L, Theiss AL (2018). Elevated d-2-hydroxyglutarate during colitis drives progression to colorectal. Proceedings of the National Academy of Sciences of the United States of America.

[ref-9] Hinshaw DC, Shevde LA (2019). The tumor microenvironment innately modulates cancer progression. Cancer Research.

[ref-10] Hänzelmann S, Castelo R, Guinney J (2013). GSVA: gene set variation analysis for microarray and RNA-seq data. BMC Bioinformatics.

[ref-11] Jafari N, Drury J, Morris AJ, Onono FO, Stevens PD, Gao T, Liu J, Wang C, Lee EY, Weiss HL, Evers BM, Zaytseva YY (2019). De novo fatty acid synthesis-driven sphingolipid metabolism promotes metastatic. Molecular Cancer Research.

[ref-12] Jiang L, Xiao L, Sugiura H, Huang X, Ali A, Kuro-o M, Deberardinis RJ, Boothman DA (2015). Metabolic reprogramming during TGFβ1-induced epithelial-to-mesenchymal. Oncogene.

[ref-13] Kazlauskas A (2015). Lysophosphatidic acid contributes to angiogenic homeostasis. Experimental Cell Research.

[ref-14] Kretschmer A, Tilki D (2017). Biomarkers in prostate cancer - Current clinical utility and future perspectives. Critical Reviews in Oncology/Hematology.

[ref-15] Kushwaha PP, Verma SS, Shankar E, Lin S, Gupta S (2022). Role of solute carrier transporters SLC25A17 and SLC27A6 in acquired resistance. Molecular Carcinogenesis.

[ref-16] Li T, Fan J, Wang B, Traugh N, Chen Q, Liu JS, Li B, Liu XS (2017a). TIMER: a web server for comprehensive analysis of tumor-infiltrating immune cells. Cancer Research.

[ref-17] Li HY, Jin N, Han YP, Jin XF (2017b). Pathway crosstalk analysis in prostate cancer based on protein-protein network data. Neoplasma.

[ref-18] Li N, Li N, Wen S, Li B, Zhang Y, Liu Q, Zheng S, Yang J, Shen L, Xing L, Zhan X (2021). HSP60 regulates lipid metabolism in human ovarian cancer. Oxidative Medicine and Cellular Longevity.

[ref-19] Li Z, Zhang H (2016). Reprogramming of glucose, fatty acid and amino acid metabolism for cancer. Cellular and Molecular Life Sciences.

[ref-20] Liu MS, Zhao H, Xu CX, Xie PB, Wang W, Yang YY, Lee WH, Jin Y, Zhou HQ (2021). Clinical significance of EPHX2 deregulation in prostate cancer. Asian Journal of Andrology.

[ref-21] Luan B, Yoon YS, Lay JL, Kaestner KH, Hedrick S, Montminy M (2015). CREB pathway links PGE2 signaling with macrophage polarization. Proceedings of the National Academy of Sciences of the United States of America.

[ref-22] Mayakonda A, Lin DC, Assenov Y, Plass C, Koeffler HP (2018). Maftools: efficient and comprehensive analysis of somatic variants in cancer. Genome Research.

[ref-23] Mendelson K, Evans T, Hla T (2014). Sphingosine 1-phosphate signalling. Development.

[ref-24] Peng Y, Xu C, Wen J, Zhang Y, Wang M, Liu X, Zhao K, Wang Z, Liu Y, Zhang T (2021). Fatty acid metabolism-related lncRNAs are potential biomarkers for predicting the overall survival of patients with colorectal cancer. Frontiers in Oncology.

[ref-25] Qi Y, Chen D, Lu Q, Yao Y, Ji C (2019). Bioinformatic profiling identifies a fatty acid metabolism-related gene risk. Disease Markers.

[ref-26] Schaeffer E, Srinivas S (2022). NCCN prostate cancer guidelines version 1.2023. https://www.nccn.org/guidelines/guidelines-detail?category=1&id=1459.

[ref-27] Shiao SL, Chu GC, Chung LW (2016). Regulation of prostate cancer progression by the tumor microenvironment. Cancer Letters.

[ref-28] Siemers NO, Holloway JL, Chang H, Chasalow SD, Ross-MacDonald PB, Voliva CF, Szustakowski JD (2017). Genome-wide association analysis identifies genetic correlates of immune. PLOS ONE.

[ref-29] Song J, Park S, Oh J, Kim D, Ryu JH, Park WC, Baek IJ, Cheng X, Lu X, Jin EJ (2020). NUDT7 loss promotes Kras(G12D) CRC development. Cancers (Basel).

[ref-30] Stephenson AJ, Scardino PT, Eastham JA, Bianco FJ, Dotan ZA, DiBlasio CJ, Reuther A, Klein EA, Kattan MW (2005). Postoperative nomogram predicting the 10-year probability of prostate cancer. Journal of Clinical Oncology.

[ref-31] Subramanian A, Tamayo P, Mootha VK, Mukherjee S, Ebert BL, Gillette MA, Paulovich A, Pomeroy SL, Golub TR, Lander ES, Mesirov JP (2005). Gene set enrichment analysis: a knowledge-based approach for interpreting. Proceedings of the National Academy of Sciences of the United States of America.

[ref-32] Sun Z, Jing C, Guo X, Zhang M, Kong F, Wang Z, Jiang S, Wang H (2021). Comprehensive analysis of the immune infiltrates of pyroptosis in kidney renal. Frontiers in Oncology.

[ref-33] Szklarczyk D, Gable AL, Nastou KC, Lyon D, Kirsch R, Pyysalo S, Doncheva NT, Legeay M, Fang T, Bork P, Jensen LJ, von Mering C (2021). The STRING database in 2021: customizable protein-protein networks, and functional characterization of user-uploaded gene/measurement sets. Nucleic Acids Research.

[ref-34] Tang Y, Li C, Zhang YJ, Wu ZH (2021). Ferroptosis-related long non-coding RNA signature predicts the prognosis of head. International Journal of Biological Sciences.

[ref-35] Tibshirani R, Bien J, Friedman J, Hastie T, Simon N, Taylor J, Tibshirani RJ (2012). Strong rules for discarding predictors in lasso-type problems. Journal of the Royal Statistical Society: Series B Stat Methodol.

[ref-36] Vriens K, Christen S, Parik S, Broekaert D, Yoshinaga K, Talebi A, Dehairs J, Escalona-Noguero C, Schmieder R, Cornfield T, Charlton C, Romero-Pérez L, Rossi M, Rinaldi G, Orth MF, Boon R, Kerstens A, Kwan SY, Faubert B, Méndez-Lucas A, Kopitz CC, Chen T, Fernandez-Garcia J, Duarte JAG, Schmitz AA, Steigemann P, Najimi M, Hägebarth A, Ginderachter JAV, Sokal E, Gotoh N, Wong KK, Verfaillie C, Derua R, Munck S, Yuneva M, Beretta L, DeBerardinis RJ, Swinnen JV, Hodson L, Cassiman D, Verslype C, Christian S, Grünewald S, Grünewald TGP, Fendt SM (2019). Evidence for an alternative fatty acid desaturation pathway increasing cancer. Nature.

[ref-37] Wise HM, Hermida MA, Leslie NR (2017). Prostate cancer, PI3K, PTEN and prognosis. Clinical Science.

[ref-38] Xiao W, Wang X, Wang T, Chen B, Xing J (2019). HAO2 inhibits malignancy of clear cell renal cell carcinoma by promoting lipid. Journal of Cellular Physiology.

[ref-39] Xue Y, Guo C, Hu F, Zhu W, Mao S (2020). PPARA/RXRA signalling regulates the fate of hepatic non-esterified fatty acids in a sheep model of maternal undernutrition. Biochimica et Biophysica Acta - Molecular and Cell Biology of Lipids.

[ref-40] Yang W, Soares J, Greninger P, Edelman EJ, Lightfoot H, Forbes S, Bindal N, Beare D, Smith JA, Thompson IR, Ramaswamy S, Futreal PA, Haber DA, Stratton MR, Benes C, McDermott U, Garnett MJ (2013). Genomics of drug sensitivity in cancer (GDSC): a resource for therapeutic. Nucleic Acids Research.

[ref-41] Yin W, Jiang X, Tan J, Xin Z, Zhou Q, Zhan C, Fu X, Wu Z, Guo Y, Jiang Z, Ren C, Tang G (2020). Development and validation of a tumor mutation burden-related immune prognostic. Frontiers in Oncology.

[ref-42] Yoshihara K, Shahmoradgoli M, Martínez E, Vegesna R, Kim H, Torres-Garcia W, Treviño V, Shen H, Laird PW, Levine DA, Carter SL, Getz G, Stemke-Hale K, Mills GB, Verhaak RG (2013). Inferring tumour purity and stromal and immune cell admixture from expression. Nature Communications.

[ref-43] Zhang C, Cheng W, Ren X, Wang Z, Liu X, Li G, Han S, Jiang T, Wu A (2017). Tumor purity as an underlying key factor in glioma. Clinical Cancer Research.

