# Supplementary figures and images for: Comprehensive analysis of fatty acid metabolism-related gene signatures for predicting prognosis in patients with prostate cancer
Source: PeerJ. 2023 Jan 10;11:e14646. doi: 10.7717/peerj.14646 (PMC9838212; doi:10.7717/peerj.14646)

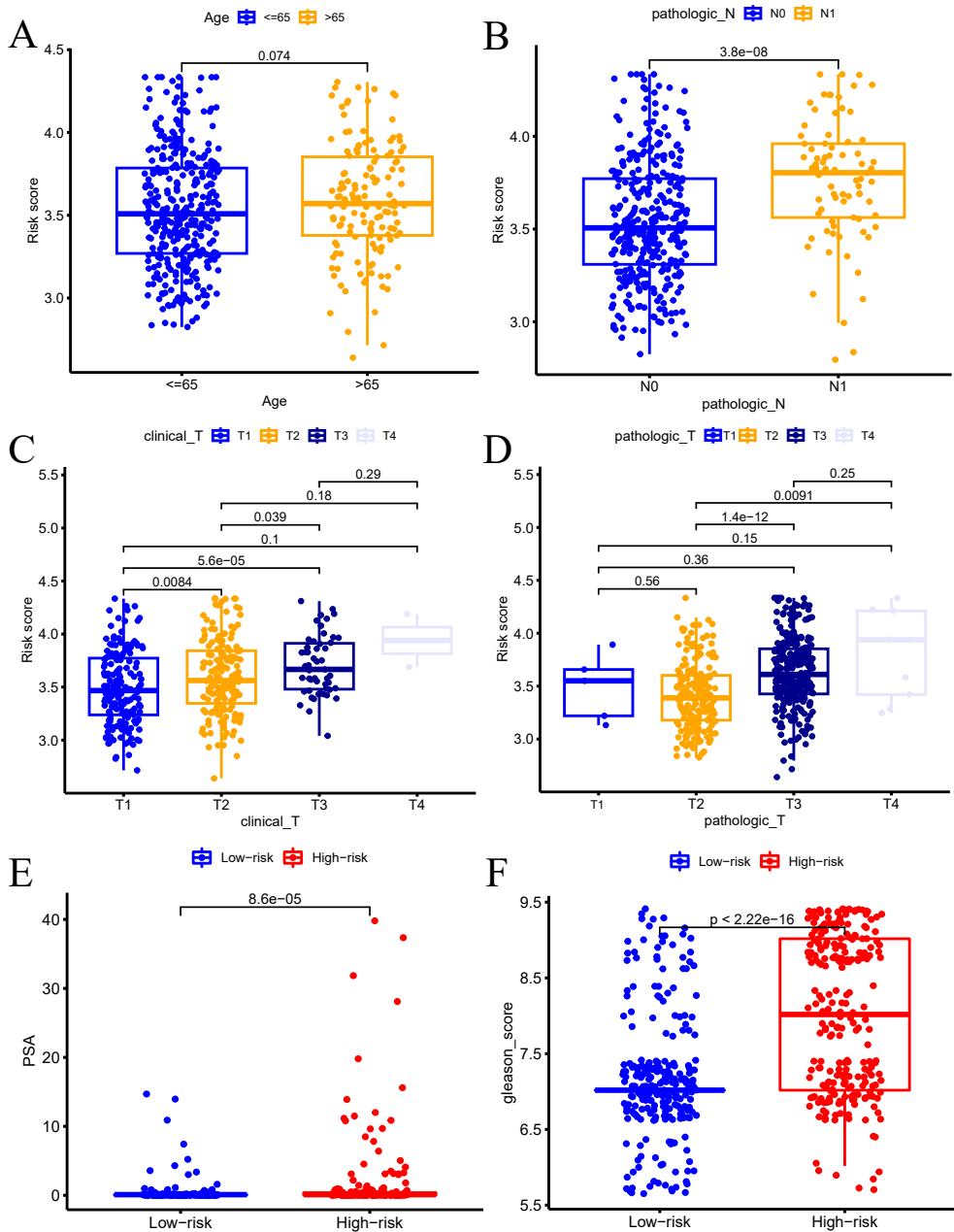

Supplement: Supplemental Information 1 [file peerj-11-14646-s001.pdf]

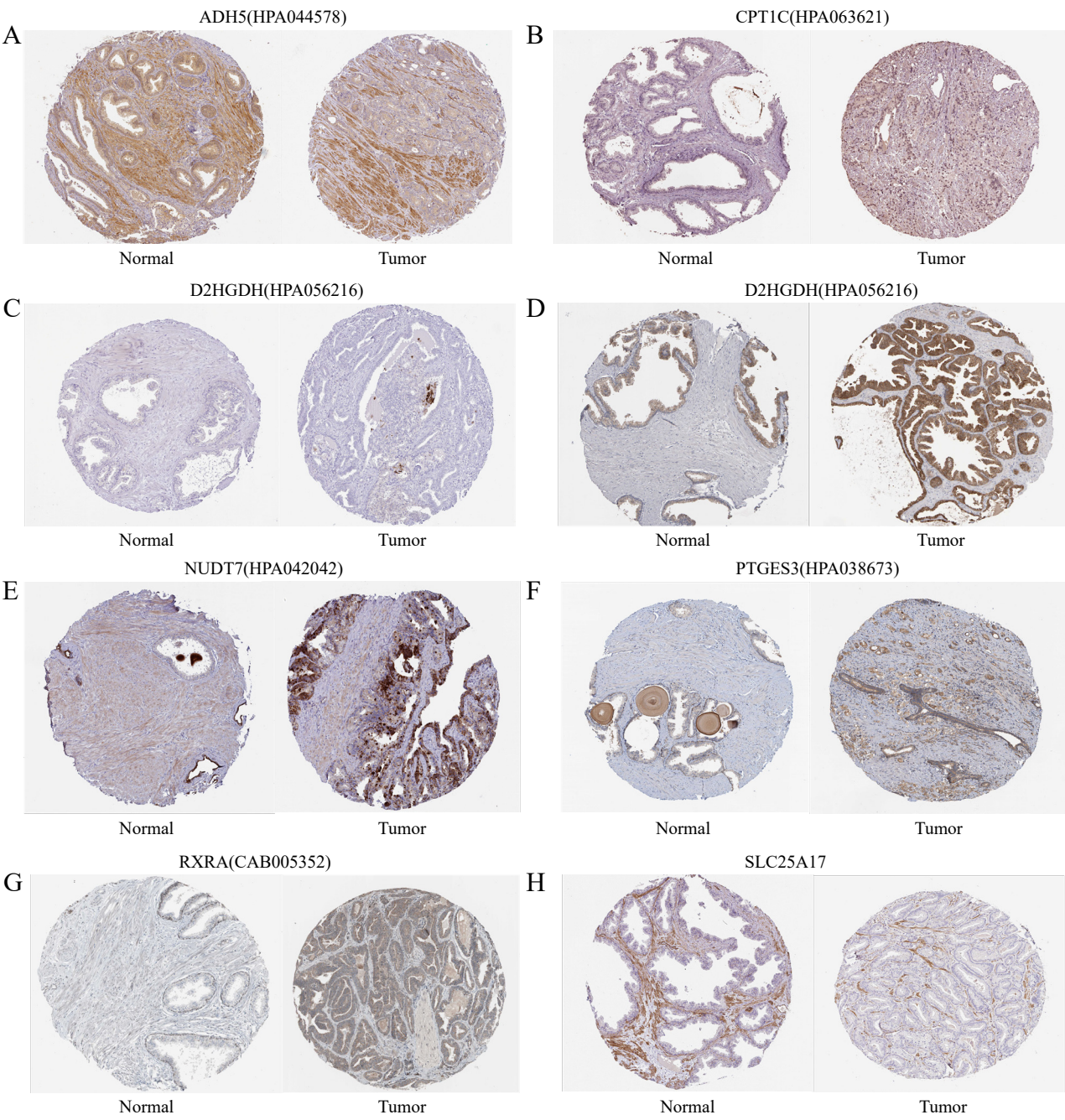

Supplement: Supplemental Information 2 [file peerj-11-14646-s002.pdf]

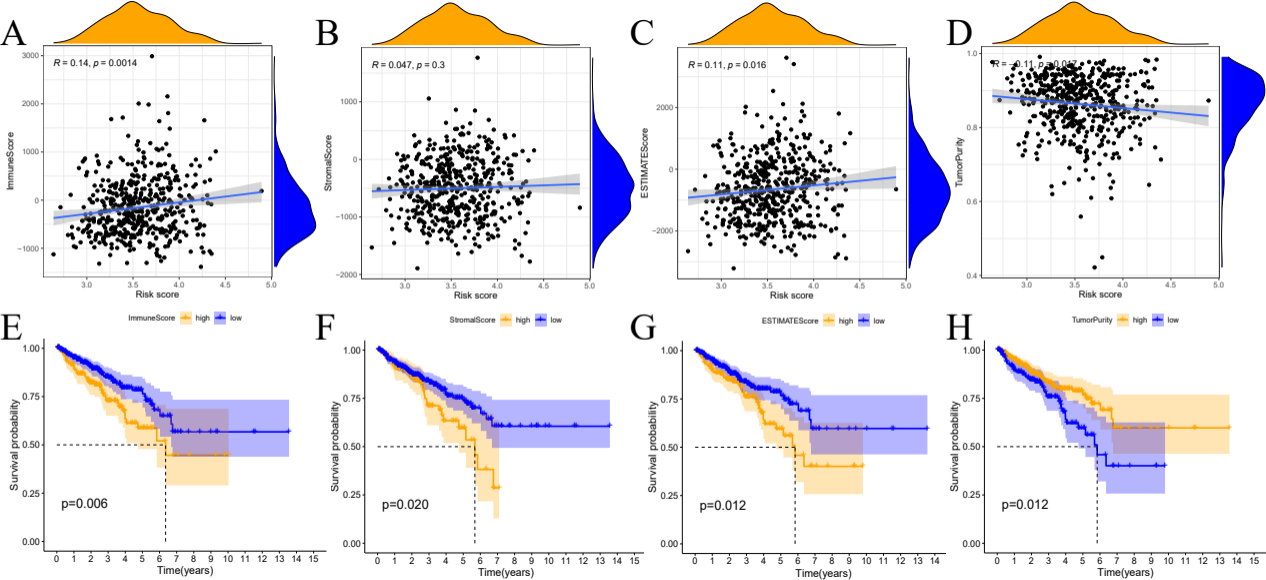

Supplement: Supplemental Information 3 [file peerj-11-14646-s003.pdf]

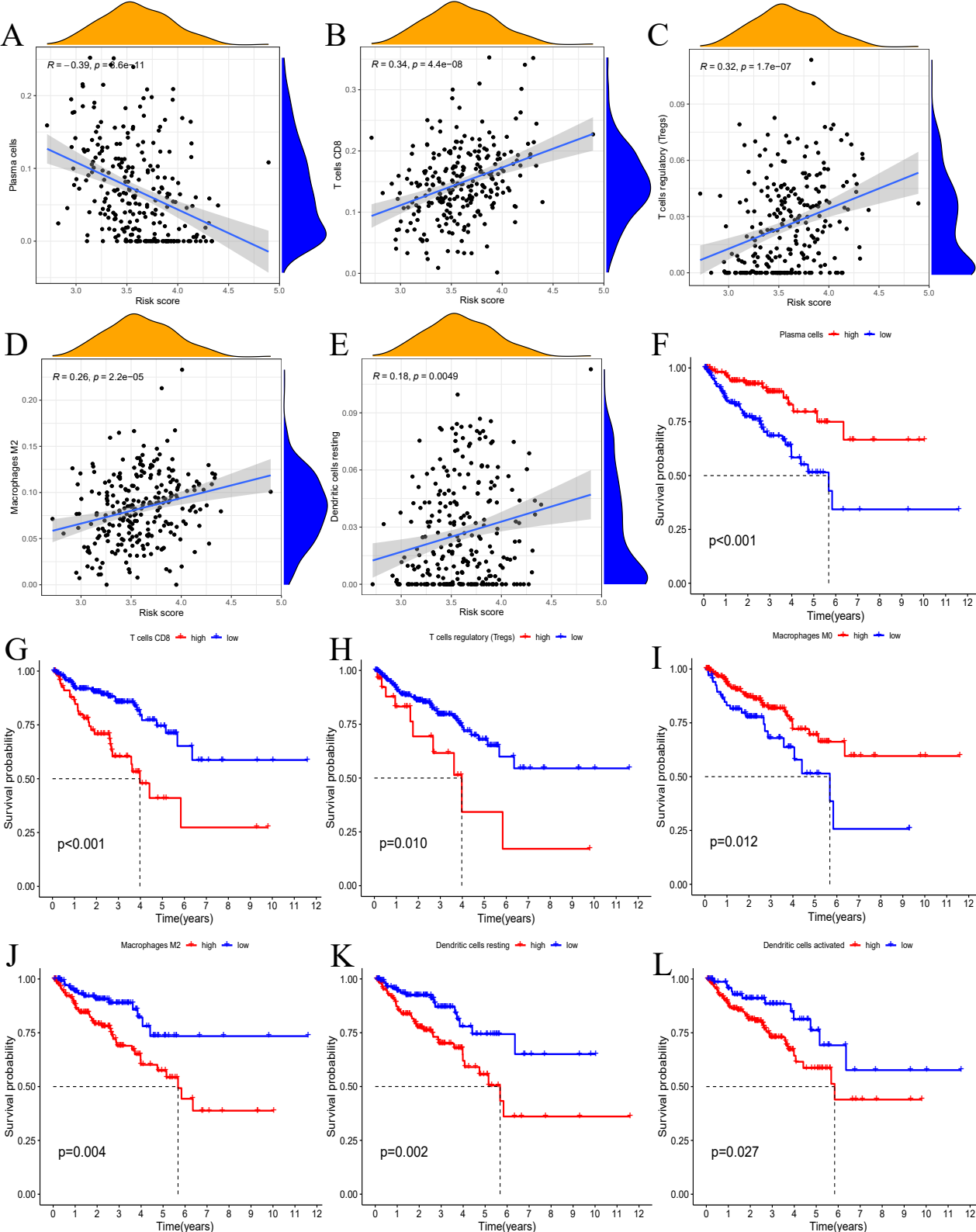

Supplement: Supplemental Information 4 [file peerj-11-14646-s004.pdf]

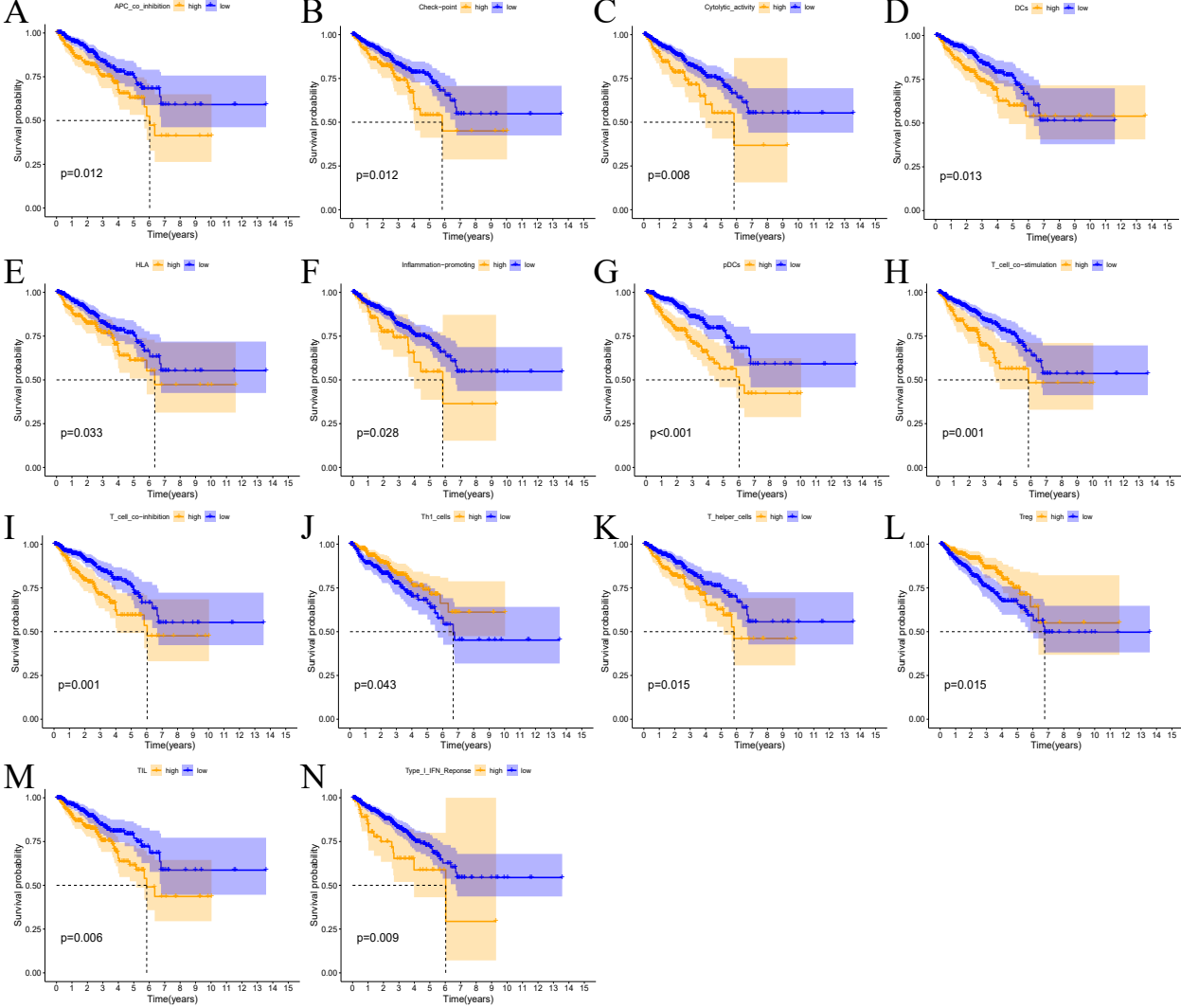

Supplement: Supplemental Information 5 [file peerj-11-14646-s005.pdf]

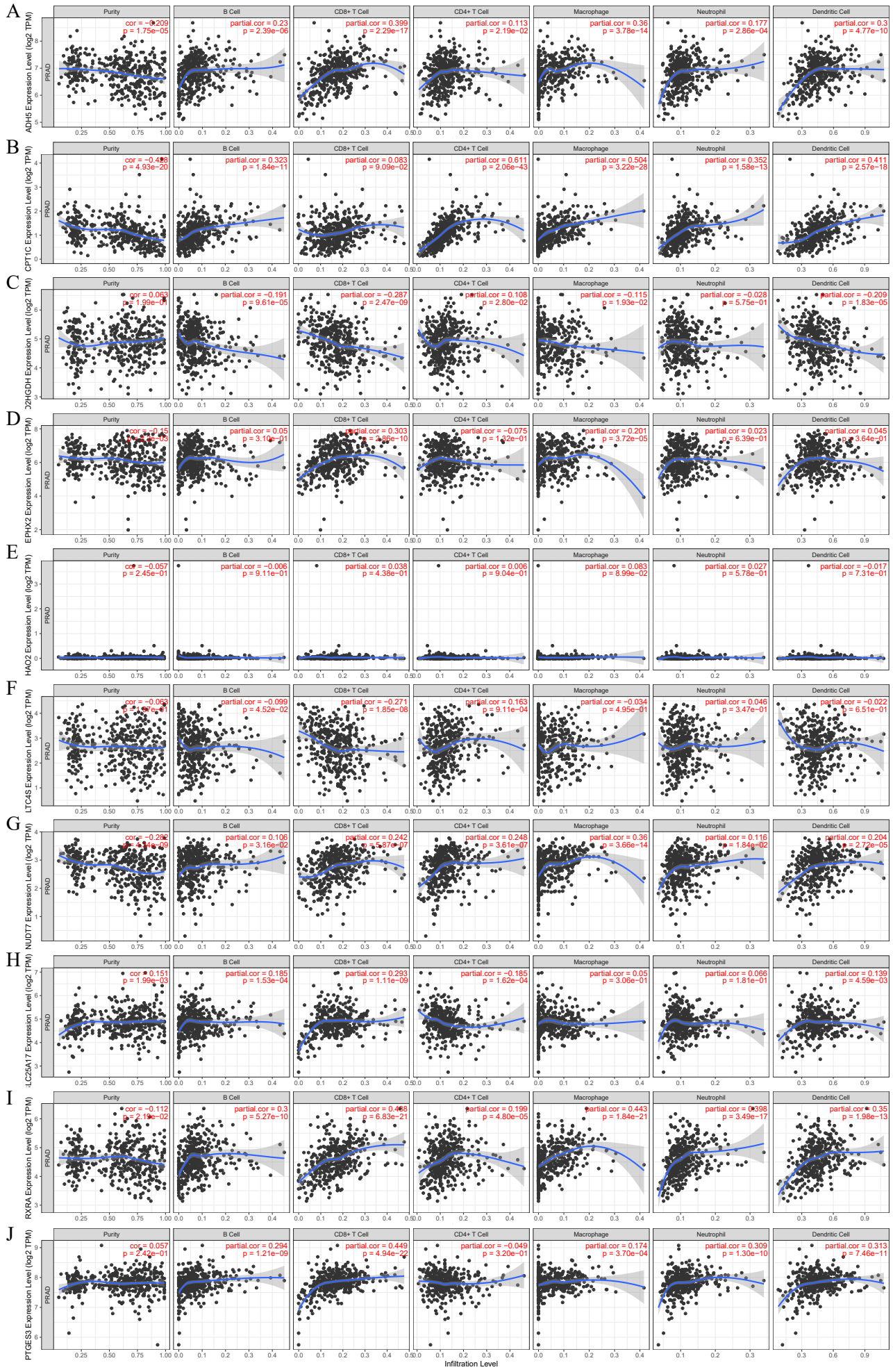

Supplement: Supplemental Information 6 — Correlation of model gene expression levels with the abundance of immune cell infiltration. [file peerj-11-14646-s006.pdf]
