# Supplementary material for: Comprehensive analysis of fatty acid metabolism-related gene signatures for predicting prognosis in patients with prostate cancer
Source: PeerJ. 2023 Jan 10;11:e14646. doi: 10.7717/peerj.14646 (PMC9838212; doi:10.7717/peerj.14646)
Supplement: Supplemental Information 7 [file peerj-11-14646-s007.docx]

Table S1. Characteristics of patients with prostate cancer based on TCGA

| **Characteristics** |  | **Number of cases** | **Percentages(%)** |
| --- | --- | --- | --- |
| Age | ≤65 | 349 | 71.37 |
|  | ＞65 | 140 | 28.63 |
| Clinical T | T1 | 174 | 35.59 |
|  | T2 | 172 | 35.18 |
|  | T3 | 51 | 10.44 |
|  | T4 | 2 | 0.4 |
|  | Unknow | 90 | 18.5 |
| Pathologic N | N0 | 340 | 69.53 |
|  | N1 | 77 | 15.75 |
|  | Unknow | 72 | 14.72 |
| Pathologic T | T2 | 185 | 37.83 |
|  | T3 | 288 | 58.9 |
|  | T4 | 9 | 1.84 |
|  | Unknow | 7 | 1.43 |
