# Supplementary material for: Comprehensive analysis of fatty acid metabolism-related gene signatures for predicting prognosis in patients with prostate cancer
Source: PeerJ. 2023 Jan 10;11:e14646. doi: 10.7717/peerj.14646 (PMC9838212; doi:10.7717/peerj.14646)
Supplement: Supplemental Information 8 [file peerj-11-14646-s008.docx]

Table S2. Characteristics of patients with prostate cancer based on GEO

| **Characteristics** |  | **Number of cases** | **Percentages(%)** |
| --- | --- | --- | --- |
| Age | ≤65 | 118 | 84.29 |
|  | ＞65 | 22 | 15.71 |
| MetSite | No | 136 | 97.14 |
|  | Yes | 4 | 2.86 |
| ClinT_Stage | T1 | 79 | 56.43 |
|  | T2 | 54 | 38.57 |
|  | T3 | 6 | 4.29 |
|  | Unknow | 1 | 0.71 |
| PathStage | T2 | 86 | 61.43 |
|  | T3 | 47 | 33.57 |
|  | T4 | 7 | 5 |
